# Stenting for symptomatic vertebral artery stenosis: The Vertebral Artery Ischaemia Stenting Trial

**DOI:** 10.1212/WNL.0000000000004385

**Published:** 2017-09-19

**Authors:** Hugh S. Markus, Susanna C. Larsson, Wilhelm Kuker, Ursula G. Schulz, Ian Ford, Peter M. Rothwell, Andrew Clifton

**Affiliations:** From the Stroke Research Group (H.S.M., S.C.L.), Department of Clinical Neurosciences, University of Cambridge, Cambridge Biomedical Campus; Nuffield Department of Clinical Neurosciences (W.K., U.G.S., P.M.R.), John Radcliffe Hospital, University of Oxford; Robertson Centre for Biostatistics (I.F.), University of Glasgow; and Department of Neuroradiology (A.C.), St. George's Hospital, London, UK.

## Abstract

**Objective:**

To compare in the Vertebral Artery Ischaemia Stenting Trial (VIST) the risks and benefits of vertebral angioplasty and stenting with best medical treatment (BMT) alone for symptomatic vertebral artery stenosis.

**Methods:**

VIST was a prospective, randomized, open-blinded endpoint clinical trial performed in 14 hospitals in the United Kingdom. Participants with symptomatic vertebral stenosis ≥50% were randomly assigned (1:1) to vertebral angioplasty/stenting plus BMT or to BMT alone with randomization stratified by site of stenosis (extracranial vs intracranial). Because of slow recruitment and cessation of funding, recruitment was stopped after 182 participants. Follow-up was a minimum of ≥1 year for each participant.

**Results:**

Three patients did not contribute any follow-up data and were excluded, leaving 91 patients in the stent group and 88 in the medical group. Mean follow-up was 3.5 (interquartile range 2.1–4.7) years. Of 61 patients who were stented, stenosis was extracranial in 48 (78.7%) and intracranial in 13 (21.3%). No periprocedural complications occurred with extracranial stenting; 2 strokes occurred during intracranial stenting. The primary endpoint of fatal or nonfatal stroke occurred in 5 patients in the stent group vs 12 in the medical group (hazard ratio 0.40, 95% confidence interval 0.14–1.13, *p* = 0.08), with an absolute risk reduction of 25 strokes per 1,000 person-years. The hazard ratio for stroke or TIA was 0.50 (*p* = 0.05).

**Conclusions:**

Stenting in extracranial stenosis appears safe with low complication rates. Large phase 3 trials are required to determine whether stenting reduces stroke risk.

**ISRCTN.com identifier:**

ISRCTN95212240.

**Classification of evidence:**

This study provides Class I evidence that for patients with symptomatic vertebral stenosis, angioplasty with stenting does not reduce the risk of stroke. However, the study lacked the precision to exclude a benefit from stenting.

Posterior circulation stroke accounts for 20% of ischemic stroke.^1^ A quarter occurs in patients with stenosis in the vertebral and/or basilar arteries.^1^ Information about optimal management is lacking compared with symptomatic carotid stenosis, for which large international trials demonstrated a benefit from endarterectomy^2^ and that stenting may be appropriate in selected patients.^3^

Patients with recently symptomatic vertebrobasilar stenosis have a high risk of recurrent stroke similar to carotid stenosis, with the highest risk in the first month.^4^ Vertebral artery (VA) stenosis can be treated with angioplasty and/or stenting.^1^ Case series have suggested that stenting may be an effective treatment option, but nonrandomized studies are subject to publication bias.^5,6^ Very low complication rates (1%–1.5%) have been reported with stenting for extracranial VA stenosis,^6^ but higher complication rates have been noted for intracranial stenosis at 7% to 10%.^5^ However, recent data from randomized trials have dampened enthusiasm. The Stenting and Aggressive Medical Management for Preventing Recurrent Stroke in Intracranial Stenosis (SAMMPRIS) trial reported worse outcome for stenting compared with best medical therapy (BMT) in patients with stenosis in a variety of intracranial cerebral arteries^7^; however, there were few patients with VA stenosis.^8^ The Vertebral Artery Stenting Trial (VAST) included patients with intracranial and extracranial VA stenosis and found no significant difference between stenting and BMT, but it was underpowered to detect a difference.^9^

The Vertebral Artery Ischaemia Stenting Trial (VIST) compared risks and benefits of angioplasty and stenting plus BMT for recently symptomatic VA stenosis with BMT alone. A meta-analysis combining results from VIST and previous trials was conducted.

## METHODS

### Study design and participants.

VIST was a prospective, randomized open-blinded endpoint clinical trial performed at 14 hospitals with specialized stroke and interventional radiology services in the United Kingdom. VIST sites and recruitment rate are shown in table e-1 at Neurology.org. The plan was to extend the study to other countries, but as a result of cessation of funding because of slower-than-anticipated recruitment, 182 of 540 patients, from the United Kingdom alone, were recruited.

Patients presenting with posterior circulation TIA or nondisabling stroke and VA stenosis resulting from presumed atheromatous disease with stenosis ≥50% were included. Other eligibility criteria were ability to consent and willingness to be randomized to either treatment; if randomized to stenting, it could be performed within 2 weeks. During recruitment of the first 100 patients, patients had to have had symptoms within the last 6 months, but this was changed to 3 months in view of data showing that stroke risk was highest in the first 3 months.

Exclusion criteria were VA stenosis caused by dissection, vertebral stenting felt to be technically impracticable (e.g., access problems), previous stenting in randomized artery, and pregnancy and lactation in women.

Before randomization, the likely presence of a VA stenosis had to be demonstrated on imaging and confirmed by 2 experienced neuroradiologists. The following imaging modalities were acceptable: magnetic resonance (MR) angiography (preferably contrast enhanced), contrast-enhanced CT angiography, and intra-arterial digital subtraction angiography (DSA). It was recommended that an additional imaging modality be used if there was any doubt about the result of a noninvasive screening test. The patient would be randomized only if the 2 methods provided concordant and appropriate results. The degree of VA stenosis was calculated by a method based on North American Symptomatic Carotid Endarterectomy Trial (NASCET) in which the residual luminal diameter (R) was divided by vessel diameter (D) at a point distal to the stenosis where normal vessel caliber was restored and applying the following formula: ([1 – R]/D) × 100 = degree of stenosis.^2^ When normal distal vessel was not available, e.g., for distal stenosis, the proximal normal artery diameter was used as the denominator, a method based on the Warfarin Aspirin Symptomatic Intracranial Disease (WASID) measurement of intracranial stenosis.^10^

### Standard protocol approvals, registrations, and patient consents.

The study was approved by Multicentre Ethics Committee in England (REC 08/H0711/2). All patients gave written informed consent.

### Randomization and masking.

Patients were randomly assigned (1:1) to vertebral angioplasty/stenting plus BMT or BMT alone by an online randomization service provided by Kings College London. To account for the differing recurrent stroke risk associated with site of VA stenosis, randomization was stratified by site of VA stenosis (V1 vs V2/V3 vs V4).^1^ Both patients and clinicians were aware of treatment allocation, but an independent adjudication committee masked to treatment allocation assessed all primary and secondary endpoints.

### Procedures.

All patients were expected to receive BMT, including antiplatelets or anticoagulation (when appropriate) and control of medical risk factors. Use of antiplatelet agents was recorded. Specific drugs to be used were not mandated.

The recommended antiplatelet therapy during the procedure was clopidogrel and aspirin with loading with clopidogrel at least 12 hours before the procedure (300–600 mg) if the patient was not already taking clopidogrel. It was recommended that clopidogrel and aspirin be continued for at least 1 month after the procedure, after which standard antiplatelet therapy for stroke prevention was used.

It was recommended that stenting, rather than angioplasty, be preferred for proximal vertebral stenosis, but for distal stenosis, the choice was at the discretion of the radiologist. Stent choice was at the discretion of the radiologist, but stents were CE marked for treatment of arterial stenosis. To be eligible to participate, a center had to have a consultant neurologist/stroke physician and a consultant interventional radiologist with experience in cerebral angioplasty/stenting. Interventionists were expected to have performed a minimum of 50 stenting procedures, of which at least 10 were on cerebral vessels.

### Follow-up.

Both entry and follow-up data were collected via an online electronic case report form. Participants allocated to stenting were seen at the time of the procedure and at 1 month and 1 year after randomization. In addition, telephone follow-up was performed at 6 months and 2 years and then yearly by the coordinating center by a designated stroke physician or neurologist using a standard form. If patients had outcome events during follow-up, an endpoint form was completed, results of imaging were obtained, and data were reviewed by the adjudication committee. Repeat imaging with either MR angiography or CT angiography at 1 year to check for vessel patency was encouraged but not mandated.

Angiographic imaging at baseline and at 12 months was assessed by central reading by an experienced neuroradiologist (A.C.). Restenosis was defined as any residual or recurrent stenosis of at least 50% or occlusion of the VA on CT or MR angiography during follow-up.

### Outcomes.

The primary endpoint was fatal or nonfatal stroke in any arterial territory (including periprocedural stroke) during follow-up. Stroke was defined as a focal neurologic deficit of presumed vascular cause, lasting >24 hours, of any severity. Secondary endpoints included stroke and TIA during follow-up, posterior circulation stroke (including periprocedural stroke) during follow-up, fatal or nonfatal stroke in any arterial territory within 90 days of randomization, and death resulting from any cause during follow-up. Periprocedural stroke was defined as stroke within 30 days of intervention.

### Sample size.

For sample size estimates, the following numbers were used: stroke risk in the medically treated arm of 24% over a 3-year period, and a risk reduction in the stented arm of 45%. The number of patients needed was estimated to be 245 per group, assuming *p* = 0.05 and 80% power. Calculations were performed with a χ^2^ test comparing 2 proportions with nQuery Advisor software version 6.02. Sample size was increased by 10% to account for crossovers or loss to follow-up to give a sample size of 540. Because of slow recruitment, support for continued recruitment by the funder was withdrawn after 182 patients were recruited; at that point, an analysis was planned after every patient had ≥1 years of follow-up.

### Statistical analysis.

The main analyses performed were on an intention-to-treat basis. We also conducted per-protocol analyses including patients who received the assigned treatment and had at least 50% VA stenosis confirmed at angiogram. Differences in baseline characteristics between treatment groups were compared by use of the *t* test for continuous variables and χ^2^ or Fisher exact tests for categorical variables.

Hazard ratios (HRs) with 95% confidence intervals (CIs) were estimated with Cox proportional hazards regression models. Each patient accrued follow-up time from the date of randomization until the time of first event of each type, death, or March 1, 2016, by which follow-up of at least 1 year was available for all patients. Absolute event rates were calculated by dividing the number of events by the number of person-years. The proportional hazards assumption was tested with scaled Schoenfeld residuals. Kaplan-Meier survival analysis was used to construct time-to-event curves, and the log-rank test was used to compare the cumulative events between groups.

### Meta-analysis.

A random-effects meta-analysis was performed to combine the results from VIST and previous trials. The search strategy for identifying previous trials is reported in the supplemental data.

All statistical tests were 2 sided. Statistical analyses were performed with Stata version 14.1 (StataCorp, College Station, TX).

## RESULTS

### Trial profile and baseline characteristics.

**Figure 1:**
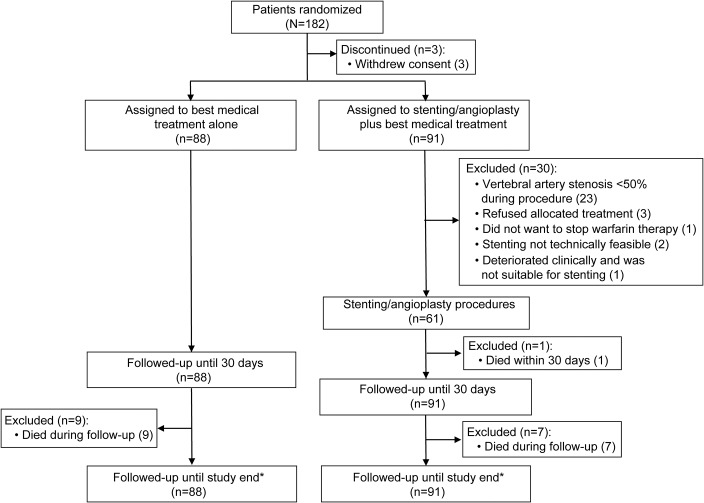
Trial profile *Intention-to-treat population.

The trial profile is shown in figure 1. Between October 23, 2008, and February 4, 2015, 182 patients were enrolled. Three patients (2 withdrew after randomization and 1 did not attend after the initial randomization visit) did not contribute any follow-up data and were excluded. None of these patients had outcome events. Of the 179 remaining, 88 were assigned to BMT alone (medical group) and 91 to stenting/angioplasty plus BMT (stent group). Follow-up data until March 2016 were available for all 179 patients.

Characteristics of patients at baseline were well matched (table 1), but time from last symptoms to randomization was shorter in the stenting arm by a mean of 12.8 days (*p* = 0.04). The percentage of patients randomized within 14 days of last symptoms was 47% in the stented and 30% in the medical arm (*p* = 0.02). The location of the VA target stenosis was extracranial in 83% and intracranial in 17%; most extracranial stenoses affected the V1 segment.

**Table 1:**
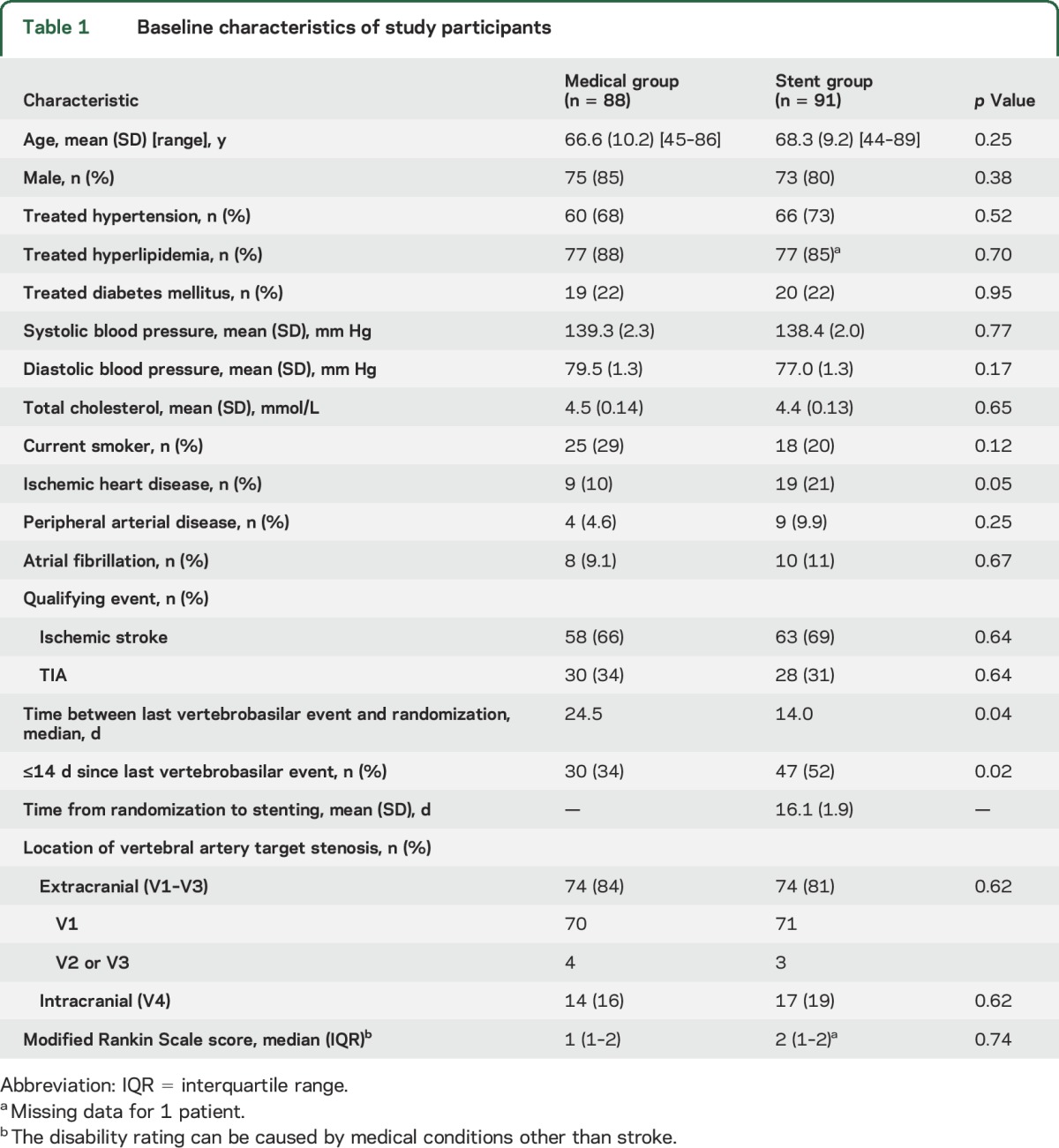
Baseline characteristics of study participants

Median follow-up was 3.5 years (interquartile range 2.1–4.7 years). Medical treatment and risk factor control at baseline and follow-up were well balanced between the 2 groups, except for slightly higher antiplatelet use in the stented arm in the first month (table 2).

**Table 2:**
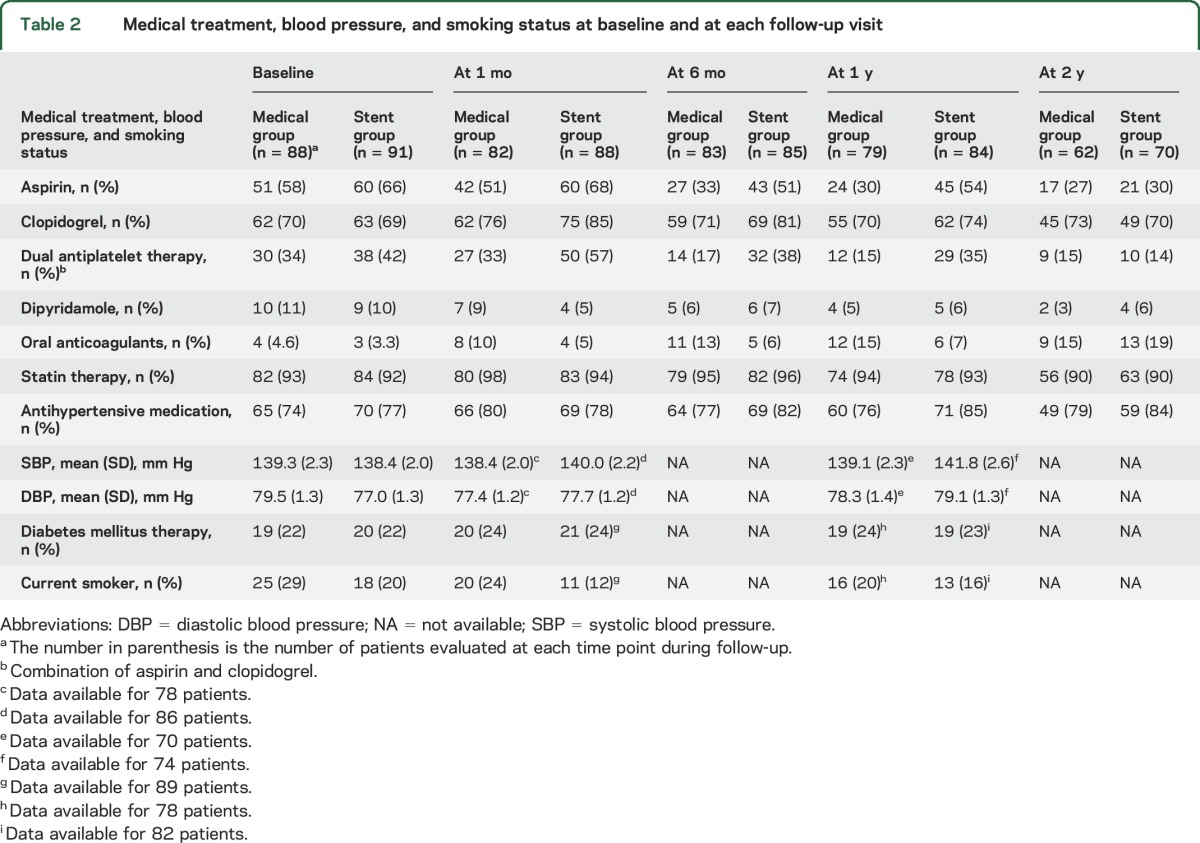
Medical treatment, blood pressure, and smoking status at baseline and at each follow-up visit

### Details of intervention.

Of 91 patients randomized to stenting, the procedure was not performed in 30 (33.0%) (figure 1). The major reason, in 23 (76.7%) participants, was the finding of stenosis <50% on DSA performed at the time of planned stenting. Of the 61 patients stented, the stenosis was extracranial in 48 (78.7%) and intracranial in 13 (21.3%). Fifty-eight (63.7%) patients had a stent (56 balloon-expandable and 2 self-expanding stents) placed, and 3 patients had angioplasty alone; no distal protection devices were used. Mean stenosis in the treated VA of stented patients was 78.7% (SD 1.6%) before stenting and 9.6% (SD 1.8%) after stenting.

There were 2 major complications during the stenting procedure, both in patients with intracranial stenosis. One died of subarachnoid hemorrhage during stenting caused by vessel rupture. A second had a nonfatal periprocedural brainstem stroke. In patients with extracranial stenosis, 1 stented patient had a nonfatal stroke within 30 days of intervention.

### Primary outcome.

**Figure 2:**
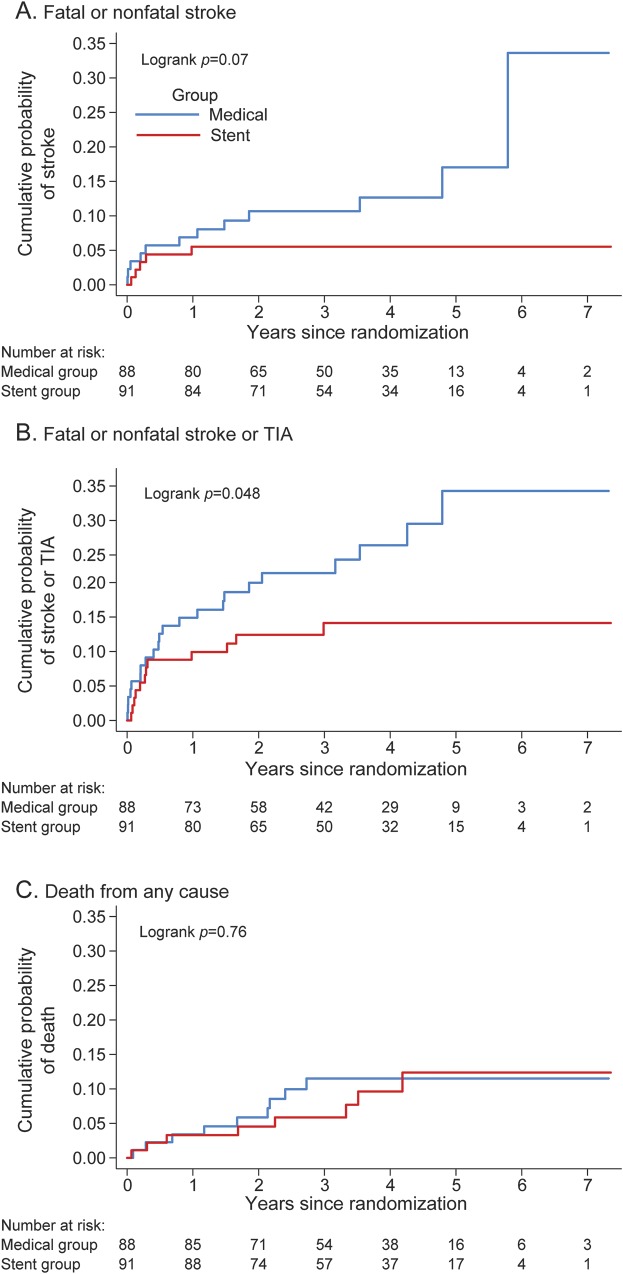
Kaplan-Meier curves Kaplan-Meier cures for the cumulative probability of (A) fatal or nonfatal stroke in any arterial territory (primary endpoint), (B) fatal or nonfatal stroke in any arterial territory or TIA, and (C) death resulting from any cause during follow-up, according to treatment group (intention-to-treat population). Log-rank test was used to test the hypothesis that stroke incidence, stroke or TIA incidence, or mortality rate between groups was the same.

The primary endpoint of fatal or nonfatal stroke occurred in 5 patients (including 1 fatal stroke) in the stent group and in 12 patients (including 2 fatal strokes) in the medical group, with an HR of 0.40 (95% CI 0.14–1.13, *p* = 0.08) (table 3 and figure 2A). For the primary endpoint, there were 41 strokes per 1,000 person-years in the medical group compared with 16 strokes per 1,000 person-years in the stent group. Therefore, the absolute risk benefit was 25 strokes per 1,000 person-years.

**Table 3:**
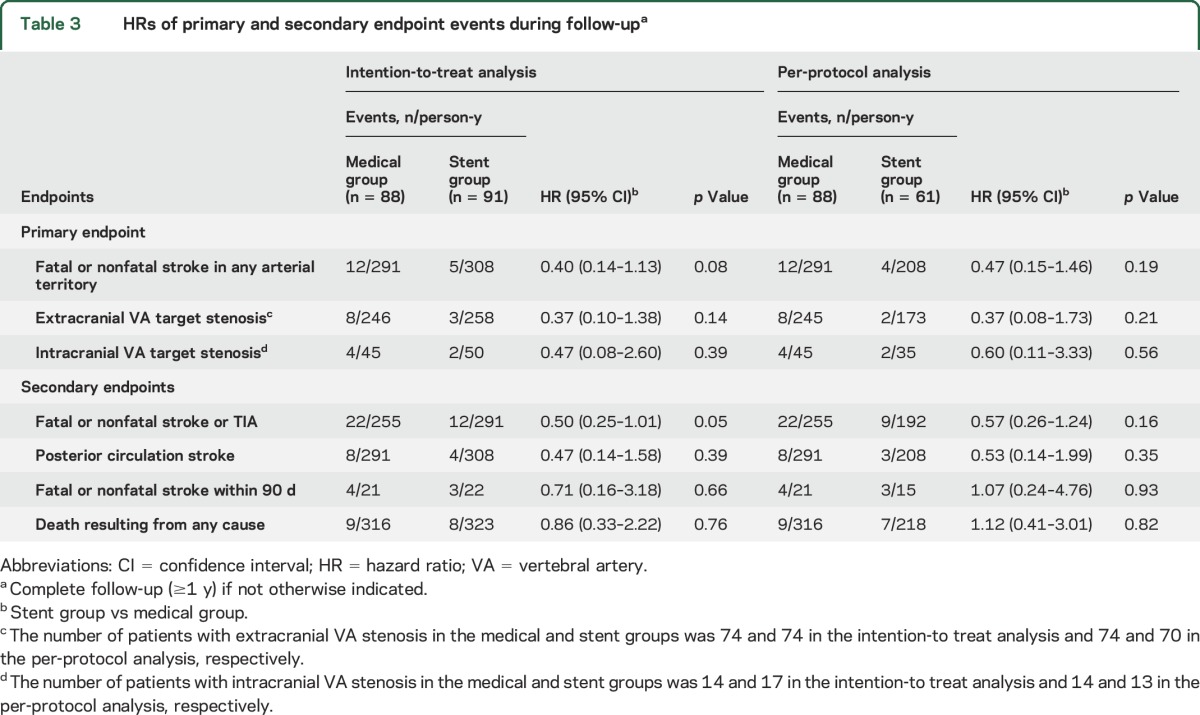
HRs of primary and secondary endpoint events during follow-up^a^

During follow-up, 1 stroke occurred among the 8 patients with atrial fibrillation in the medical group. No strokes occurred among the 10 patients with atrial fibrillation in the stent group.

As a result of the imbalance in time from symptoms to randomization between groups, an exploratory post hoc analysis was performed with adjustment for days from last symptoms (i.e., last vertebrobasilar TIA or stroke) to randomization. The corresponding HR for the primary endpoint was 0.34 (95% CI 0.12–0.98, *p* = 0.046). In addition, a second post hoc analysis in patients randomized within 2 weeks after the last symptom was performed; the HR of the primary endpoint was 0.30 (95% CI 0.09–0.99, *p* = 0.048; medical group 8 of 30, stent group 4 of 47).

### Key secondary outcomes.

For the composite secondary endpoint of fatal or nonfatal stroke or TIA, the HR was 0.50 (95% CI 0.25–1.01, *p* = 0.05) (table 3 and figure 2B). The HR in patients with extracranial and intracranial VA stenosis was 0.37 (95% CI 0.10–1.36) and 0.47 (95% CI 0.08–2.60), respectively (table 3). Other secondary endpoints, fatal or nonfatal stroke within 90 days and death resulting from any cause (figure 2C), did not differ between the 2 groups (table 3). The per-protocol analyses yielded similar results (table 3).

### Adverse events.

Adverse events are listed in table e-2. There was no difference between the 2 treatment groups.

### Follow-up angiographic imaging.

Follow-up angiographic imaging with CT or MR angiography was performed in 47 of 61 participants undergoing stenting. This showed 3 stent occlusions, 2 in V1 and 1 in V2; 2 patients were asymptomatic, and 1 participant had a TIA.

## DISCUSSION

VIST is the largest RCT comparing stenting plus BMT with BMT alone in patients with symptomatic VA stenosis. Stenting, particularly for extracranial stenosis, appeared safe. No significant difference was found in risk of stroke between the 2 treatment arms. Over a median follow-up of 3.5 years, the stented group had a nonsignificant 60% lower risk for the primary endpoint of fatal and nonfatal stroke compared with the BMT group. A similar magnitude of risk reduction was seen for posterior circulation stroke alone and for the combined endpoint of stroke and TIA.

Natural history data have shown that the risk of recurrent stroke after TIA or minor stroke due to vertebral stenosis is much greater in the first days and few weeks after the event.^4^ This is a pattern similar to that seen in symptomatic carotid stenosis.^2^ Therefore, any intervention is likely to have greater benefit if administered early. Consistent with this, an analysis of patients randomized within 2 weeks of last symptoms showed a significant treatment benefit.

Four previous RCTs have compared stenting and/or angioplasty with medical therapy in patients with VA stenosis. The Carotid and Vertebral Artery Transluminal Angioplasty Study (CAVATAS) randomized 16 patients with extracranial VA stenosis to angioplasty or medical therapy; no stroke endpoints occurred in either group.^11^ SAMMPRIS randomized patients with a variety of intracranial stenoses to stenting or BMT.^7^ Only 60 (13%) patients had intracranial VA stenosis, and among them, the 2-year primary event rate was 9.5% in the medical group and 21.1% in the stenting group.^8^ The Vitesse Intracranial Stent Study for Ischemic Therapy (VISSIT) randomized 112 patients with symptomatic intracranial stenosis in a variety of intracerebral arteries to stenting or BMT and reported a risk similar to that in SAMMPRIS, but the number with VA stenosis was not documented.^12^ VAST randomized 115 patients (83% with extracranial stenosis) and showed a nonsignificantly higher risk of early outcomes at 30 days in the stented group but similar risk of any stroke in both groups after a median follow-up of 3 years.^9^ A meta-analysis of SAMMPRIS, VAST, and VIST showed no advantage for stenting/angioplasty vs BMT alone in extracranial and intracranial VA stenosis combined or in extracranial or intracranial VA stenosis (figure e-1).

Previous observational studies have shown that the risk of stenting is low with extracranial VA stenosis, on the order of 1%, compared with 7% to 10% for intracranial stenosis.^5,6^ However, natural history data have shown that the risk of early recurrent stroke is higher for intracranial stenosis.^4^ For this reason, it is possible that the treatment benefits vary in the 2 locations. Therefore, randomization was stratified according to location of stenosis. Because the majority of patients in VIST had extracranial stenosis, drawing firm conclusions on benefit in intracranial stenosis is difficult, although the risk of periprocedural stroke appeared higher for intracranial stenosis and the results are in line with those from SAMMPRIS showing higher risk in stented than in medically treated patients.

Strengths of VIST include the randomized design, that it is the largest study of stenting for VA stenosis, and that no patients were lost to follow-up. A criticism is that recruitment was stopped because of funding issues before the planned number of patients had been recruited. A further consideration is the intensity of medical treatment in the 2 arms. There was no difference in the use of statins or antihypertensive agents between the 2 arms. There was a slightly increased use of antiplatelet agents at the first-month follow-up in the stented arm, but it was small, reflecting the advice given that both groups should be given intensive medical therapy. An additional consideration is the high proportion of patients in the stenting arm who were found not to have stenosis >50% on DSA at the time of stenting. As a result of the small size of the vertebral arteries, noninvasive imaging with CT or MR angiography is more challenging.^1^ However, previous studies comparing these 2 noninvasive modalities with intra-arterial DSA have shown high sensitivity and specificity.^13^ Central review of entry imaging failed to confirm stenosis in approximately half of the cases in which stenosis was not confirmed, while in others, entry imaging was adjudged to be of insufficient quality to confirm stenosis. This suggests that misdiagnosis was in some cases due to radiologic interpretation, and the misdiagnosis rate appeared higher in certain centers. This finding emphasizes the need for very careful ongoing quality control with rapid review of imaging throughout the trial. A further consideration is the experience of neurointerventionists who had to perform only 10 previous procedures to enter the study.

Recruitment into the trial was slower than expected, and rates varied widely between centers. This partly reflects the fact that in the United Kingdom not all patients with posterior circulation stroke receive CT or MR angiography. This would be likely to change if further data show that stenting is associated with improved outcome. The intention was to extend the trial to overseas centers, but this was delayed because of regulatory and contract issues and sites were not opened before the funder withdrew funding.

Stenting of extracranial symptomatic vertebral stenosis was performed in this multicenter study with a low periprocedural risk and appears safe compared with BMT alone. There was a nonsignificant reduction in recurrent stroke risk in the stented compared with the BMT arm. As a result of early termination of recruitment, the projected sample size was not reached, and larger trials are now required to confirm this finding.

## Supplementary Material

Data Supplement

Coinvestigators

Accompanying Editorial

## GLOSSARY

BMT: best medical therapy
CAVATAS: Carotid and Vertebral Artery Transluminal Angioplasty Study
CI: confidence interval
DSA: digital subtraction angiography
HR: hazard ratio
MR: magnetic resonance
NASCET: North American Symptomatic Carotid Endarterectomy Trial
SAMMPRIS: Stenting and Aggressive Medical Management for Preventing Recurrent Stroke in Intracranial Stenosis
VA: vertebral artery
VAST: Vertebral Artery Stenting Trial
VISSIT: Vitesse Intracranial Stent Study for Ischemic Therapy
VIST: Vertebral Artery Ischaemia Stenting Trial
WASID: Warfarin Aspirin Symptomatic Intracranial Disease
