# Supplementary material for: Stenting for symptomatic vertebral artery stenosis: The Vertebral Artery Ischaemia Stenting Trial
Source: Neurology. 2017 Sep 19;89(12):1229–36. doi: 10.1212/WNL.0000000000004385 (PMC5606920; doi:10.1212/WNL.0000000000004385)
Supplement: Data Supplement [file supp_WNL.0000000000004385_VIST_Supplement.pdf]

ONLINE DATA SUPPLEMENT

Stenting for Symptomatic Vertebral Artery Stenosis

The Vertebral artery Ischaemia Stenting Trial

Table of content

Table e-1. Rate of recruitment by UK site 2

Table e-2. Adverse events 3

Figure e-1. Relative risk of stroke among patients randomized to stenting/angioplasty vs. best medical treatment alone in randomized trials of vertebral artery stenosis 4

Text e-1. Search strategy for systematic review 4

Table e-1. Rate of recruitment by UK site

Hospital	No. of recruited patients	No. of years of recruitment	No. of patients recruited per year
St Georges University Hospitals NHS Foundation Trust	77	7.35	10.48
Oxford University Hospitals NHS Trust	38	5.71	6.65
University Hospitals of North Midlands NHS Trust	14	4.87	2.87
Sheffield Teaching Hospitals NHS Foundation Trust	11	4.94	2.23
The Walton Centre NHS Foundation Trust	8	6.74	1.19
King's College Hospital NHS Trust	8[a]	3.33	2.40
Newcastle upon Tyne Hospitals NHS Foundation Trust	7	5.19	1.35
University College Hospitals NHS Foundation Trust	6[b]	4.44	1.35
Imperial College Healthcare NHS Trust	4	3.46	1.16
Cambridge University Hospitals NHS Foundation Trust	2	1.52	1.32
North Bristol NHS Trust	2	3.29	0.61
Leeds Teaching Hospital NHS Trust	2	3.85	0.52
Nottingham University Hospital NHS Trust	2	1.38	1.45
Royal Preston Hospital	1	2.95	0.34

[a] One of these patients withdrew after randomization.

[b] Two of these patientss withdrew after randomization.

Table e-2. Adverse events[a]

	Medical group (n = 88)	Stent group (n = 91)
	No. (%) of events	
**Adverse events, any**	**29 (33.0)**	**34 (37.4)**
**Cardiovascular events, any**	**6 (6.9)**	**7 (7.7)**
Angina	1 (1.1)	2 (2.2)
Atrial fibrillation	2 (2.3)	1 (1.1)
Brachial artery dissecting aneurysm	0 (0)	1 (1.1)
Cardiac failure	1 (1.1)	2 (2.2)
Coronary artery bypass graft	1 (1.1)	0 (0)
Myocardial infarction	0 (0)	1 (1.1)
Ventricular tachycardia	1 (1.1)	0 (0)
**Other events, any**	**23 (26.1)**	**27 (29.7)**
Abdominal pain	1 (1.1)	1 (1.1)
Acoustic neuroma	0 (0)	1 (1.1)
Acute kidney injury	1 (1.1)	0 (0)
Bone fracture	0 (0)	2 (2.2)
Cancer	0 (0)	1 (1.1)
Cervical spondylosis	1 (1.1)	0 (0)
Chest infection	5 (5.7)	4 (4.4)
Chest pain non-ischemic	1 (1.1)	2 (2.2)
Collapse, unknown cause	0 (0)	2 (2.2)
Confusion, unknown cause	1 (1.1)	0 (0)
Dizziness	1 (1.1)	0 (0)
Dizziness and vomiting	0(0)	2 (2.2)
Diarrhoea and vomiting	0 (0)	1 (1.1)
Epididymitis	0 (0)	1 (1.1)
Fall	1 (1.1)	2 (2.2)
Gastrointestinal bleeding	1 (1.1)	1 (1.1)
Grip haematoma	0 (0)	1 (1.1)
Hemicolectomy	1 (1.1)	0 (0)
Multiple sclerosis	1 (1.1)	0 (0)
Numbness	1 (1.1)	0 (0)
Pancreatitis	2 (2.3)	0 (0)
Pain during stenting	0 (0)	1 (1.1)
Presyncope, unknown cause	1 (1.1)	0 (0)
Psychosis	1 (1.1)	0 (0)
Road traffic accident	0 (0)	1 (1.1)
Sciatica	1 (1.1)	0 (0)
Seizure	1 (1.1)	1 (1.1)
Unconsciousness following stenting	0 (0)	1 (1.1)
Urinary retention	0 (0)	2 (2.2)
Urinary tract infection	1 (1.1)	0 (0)

[a] Data are No. (%). Patients could have more than one adverse event.

Figure e-1. Relative risk of stroke among patients randomized to stenting/angioplasty vs. best medical treatment alone in randomized trials of vertebral artery stenosis

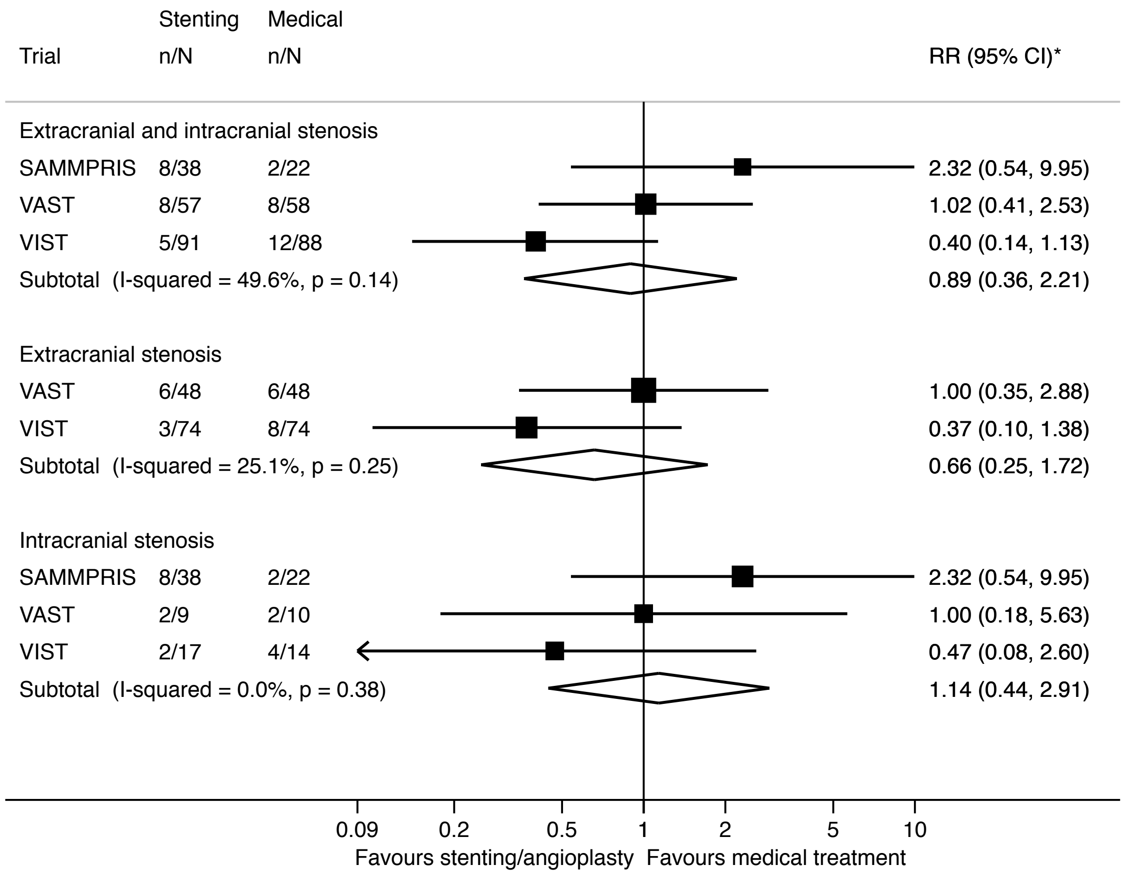

n, number of strokes; N, total number of patients; RR, relative risk; SAMMPRIS, Stenting and Aggressive Medical Management for Preventing Recurrent stroke in Intracranial Stenosis; VAST, Vertebral Artery Stenting Trial; VIST, Vertebral artery Ischaemia Stenting Trial. The results of CAVATAS are not included because there were no endpoints in either arm of the trial. Squares represent trial-specific RRs (size of the square reflects the trial-specific statistical weight); horizontal lines represent 95% confidence intervals (CIs); diamonds represent the combined RR with its 95% CI from a random-effects meta-analysis. *The RR estimates are odds ratios in SAMMPRIS and VAST, and hazard ratios in VIST.

Text e-1. Search strategy for systematic review. Randomized trials investigating the effect of stenting and/or angioplasty on recurrent stroke or TIA in symptomatic vertebral artery stenosis patients were identified by searching PubMed until March 2016. The search terms used were: (stenting OR angioplasty OR stent) AND (vertebral artery stenosis OR extracranial stenosis OR intracranial stenosis) AND (stroke OR transient ischemic attack) AND (randomized trial OR randomized controlled trial OR clinical trial).
