# Supplementary material for: Stenting for symptomatic vertebral artery stenosis: The Vertebral Artery Ischaemia Stenting Trial
Source: Neurology. 2017 Sep 19;89(12):1229–36. doi: 10.1212/WNL.0000000000004385 (PMC5606920; doi:10.1212/WNL.0000000000004385)
Supplement: Coinvestigators [file supp_WNL.0000000000004385_Coinvestigators.docx]

**COINVESTIGATORS**

**VIST Investigators**

Hugh Markus, DM, Professor (University of Cambridge; Principal Investigator, Trial Steering Committee, Writing Committee); Peter Rothwell, FMedSci, Professor (University of Oxford; Grant Holders’ Oversight Committee, Writing Committee); Andrew Clifton, FRCR (St George's Hospital; Grant Holders’ Oversight Committee, Study Neuroradiologist, Lead extracranial stenting, Writing Committee); John Bamford, MD, FRCP (University of Leeds; Trial Steering Committee, Independent Chair); Gavin Young, MD (James Cook University Hospital; Trial Steering Committee, independent member); Thompson Robinson, MD, Professor (Leicester Royal Infirmary; Trial Steering Committee, Independent member); Wilhelm Kuker, FRCR (University of Oxford; Grant Holders’ Oversight Committee, Study Neuroradiologist, Lead intracranial stenting); Ursula Schulz, DPhil (University of Oxford; Grant Holders’ Oversight Committee); Ian Ford, PhD, Professor (University of Glasgow; Grant Holders’ Oversight Committee, Statistical design); Caroline Murphy, Ms (King's College London; Grant Holders’ Oversight Oversight Committee); John Dennis, Mr (Steering Committee, Patient representative); Kath Pasco, MD (Royal Surrey County Hospital, Adjudication Committee); Ajay Bhalla, MD, FRCP (Guy’s and St Thomas’ Hospitals; Adjudication Committee); Kirsty Harkness, MD (Royal Hallamshire Hospital; Adjudication Committee); Susanna Larsson, PhD (University of Cambridge; Writing Committee, Statistical design and analysis); Melina Willson, PhD (St George’s, University of London; Study Co-coordinator); Cara Hicks,  BSc  (St George’s, University of London; Study Co-coordinator); Jennifer Lennon, BSc (St George’s, University of London; Study Co-coordinator); Lindsay Davies, PhD (University of Cambridge; Study Co-coordinator); Usman Khan, BM, BCh, PhD (St George's Hospital London; Follow-up); Robert Hurford, BM, BCh (Addenbrookes Hospital Cambridge; Follow-up); Loes Rutten-Jacobs, PhD (University of Cambridge; Interim analysis); Peter Sandercock, MD, Professor (University of Edinburgh; Data Monitoring Committee, Chair); Catriona Graham, Ms (University of Edinburgh; Data Monitoring Committee); Robin Sellar, MD, Professor (University of Edinburgh; Data Monitoring Committee).
