# Supplementary material for: Stenting for symptomatic vertebral artery stenosis: The Vertebral Artery Ischaemia Stenting Trial
Source: Neurology. 2017 Sep 19;89(12):1229–36. doi: 10.1212/WNL.0000000000004385 (PMC5606920; doi:10.1212/WNL.0000000000004385)
Supplement: Accompanying Editorial [file supp_89_12_1229_v3_index.html]

Accompanying Editorial 

# Stenting for symptomatic vertebral artery stenosis

## Accompanying Editorial

**Neurology® data supplements are not copyedited before publication. Published editorials and translations have been copyedited.  
 © 2017 American Academy of Neurology.  
  
 Files in this Data Supplement:**

- Accompanying Editorial - PDF
