# Supplementary material for: Stenting for symptomatic vertebral artery stenosis: The Vertebral Artery Ischaemia Stenting Trial
Source: Neurology. 2017 Sep 19;89(12):1229–36. doi: 10.1212/WNL.0000000000004385 (PMC5606920; doi:10.1212/WNL.0000000000004385)
Supplement: Accompanying Editorial [file supp_WNL.0000000000004385_1204.pdf]

Vertebral artery stenosis

The hurdles of stenting are too high

Annette Compter, MD, PhD
Seemant Chaturvedi, MD

Correspondence to Dr. Compter: a.compter@nki.nl

Neurology® 2017;89:1204–1205

Up to a quarter of patients with vertebrobasilar ischemic stroke or TIA have a symptomatic stenosis of the vertebrobasilar arteries.[1,2] Patients with vertebral artery (VA) stenosis >50% have a high risk of recurrent stroke, comparable to patients with symptomatic carotid artery stenosis, with the highest risk during the first weeks after the initial TIA or ischemic stroke.[3] Endovascular treatment of symptomatic VA stenosis has appeal as a treatment option that is widely performed, despite the scarcity of evidence for its safety or benefit.

In this issue of *Neurology*®, Markus et al.[1] present the final results of the Vertebral Artery Ischaemia Stenting Trial (VIST), a randomized controlled trial comparing best medical treatment alone vs best medical treatment plus stenting for symptomatic VA stenosis ≥50%. After inclusion of 182 of the 540 patients planned, the sponsor stopped recruitment because of slow recruitment and discontinuation of funding. During a mean follow-up of 3.5 years, the primary endpoint of fatal or nonfatal stroke occurred in 5 (5%) of 91 patients in the stent group and in 12 (14%) of 88 patients in the medical treatment group (hazard ratio 0.40; 95% confidence interval 0.14–1.13, $p = 0.08$). Of the 61 patients who underwent a stenting procedure, 3 patients had a periprocedural stroke within 30 days after intervention, 2 of which occurred in the 13 patients who underwent intracranial stenting. Stenting did not reduce the risk of stroke within the territory of the stenotic vessel.

Although VIST, the largest trial on symptomatic VA stenting, has its merits, the trial's methodologic shortcomings deserve discussion. First, recruitment of patients in VIST was stopped prematurely because of slow recruitment and funding issues. This inherently leads to a lack of power to detect a potential benefit of stenting over medical treatment. Diagnostic inaccuracy constitutes another major limitation of VIST: 23 (25%) of the 91 patients allocated stenting did not receive a stent because the degree of VA stenosis on digital subtraction angiography at the time of planned stenting was <50%, despite noninvasive imaging (CT angiography or magnetic resonance angiography) suggesting VA stenosis ≥50%. The inclusion of a high proportion of patients with nonsignificant VA stenosis probably resulted in a lower risk of recurrent stroke and TIA in both the stent and medical treatment groups. Lessons learned from the experience with noninvasive imaging modalities used in VIST, including the presumed location of VA stenosis, and the composition of the team interpreting the imaging results, should inform both clinical practice and future studies. Prior studies of intracranial stenting have required conventional angiography to avoid the limited accuracy of noninvasive imaging modalities.[4]

Because the highest risk for recurrent stroke occurs in the first weeks, any trial of secondary prevention in symptomatic VA stenosis should have a short interval between the initial event and enrollment. Initially, VIST included patients with symptoms of vertebrobasilar ischemia in the previous 6 months. During the course of the trial, the window for enrollment changed to within 3 months of symptoms. Overall, 47% in the stent group and 30% in the medical treatment group were randomized within 14 days of last symptoms ($p = 0.02$). Post hoc analyses adjusting for the number of days between the last event and randomization suggested a benefit for stenting over best medical treatment ($p = 0.04$). The mean interval between randomization and stenting was 16 days, which misses the high-risk period for many patients. On the other hand, the periprocedural stroke risk of endovascular treatment might be higher in the first weeks due to plaque instability in the acute phase.

Imbalance in medical therapy raises another concern with the VIST trial. Patients randomized to stenting in VIST received dual antiplatelet treatment, consisting of clopidogrel and aspirin for at least 1 month after stenting. At 1, 6, and 12 months after inclusion, more patients in the stenting group than the medical group received aspirin and clopidogrel. Results of the Clopidogrel in High-Risk Patients with Acute Nondisabling Cerebrovascular Events (CHANCE) trial suggest that combined treatment with clopidogrel and aspirin in the acute phase after

See page 1229

From the Department of Neuro-oncology (A.C.), Netherlands Cancer Institute/Antoni van Leeuwenhoek; Department of Neurology (A.C.), MC Slotervaart, Amsterdam, the Netherlands; and Department of Neurology & Stroke Program (S.C.), University of Miami Miller School of Medicine, FL.

Go to Neurology.org for full disclosures. Funding information and disclosures deemed relevant by the authors, if any, are provided at the end of the editorial.

© 2017 American Academy of Neurology
© 2017 American Academy of Neurology. Unauthorized reproduction of this article is prohibited.

TIA or ischemic stroke reduces the risk of recurrent stroke compared with aspirin alone.[5] Therefore, the higher number of patients on clopidogrel and aspirin might partly explain the nonsignificant reduction of stroke risk in the stenting group.

No further trials allocating patients with symptomatic VA stenosis to medical treatment or stenting are on the horizon. Based on the results of VIST, the Vertebral Artery Stenting Trial (VAST), and the Carotid and Vertebral Artery Transluminal Angioplasty Study (CAVATAS), stenting of extracranial VA stenosis appears relatively safe, with a low overall periprocedural stroke risk around 2%.[6,7] However, stenting plus medical treatment did not reduce the risk of recurrent stroke compared with medical treatment in the meta-analysis performed by Markus et al. Therefore, stenting of extracranial VA stenosis should not be routine clinical practice in patients with symptomatic extracranial VA stenosis. Extracranial VA stenting should only be considered in individual patients having recurrent symptoms despite optimal medical treatment.

The results of VIST, and the meta-analyses of VIST and previous trials on intracranial stenting, add to the accumulating evidence that stenting of intracranial VA stenosis is associated with a high periprocedural stroke risk around 20%,[4,8,9] without long-term benefit over medical treatment.[8,10] Results of the Stenting and Aggressive Medical Management for the Prevention of Recurrent Stroke in Intracranial Stenosis (SAMMPRIS) trial suggest that aggressive medical treatment is preferred over stenting in symptomatic intracranial artery stenosis.[10]

VIST illustrates the many pitfalls of trials evaluating endovascular therapy for stroke prevention. Following the trial's completion, high-quality evidence to justify vertebral artery stenting remains elusive.

STUDY FUNDING

No targeted funding reported.

DISCLOSURE

Annette Compter is on the executive committee of the Vertebral Artery Stenting Trial (VAST), which was supported by the Dutch Heart Foundation (2007B045). Seemant Chaturvedi is on the executive committee of the CREST 2 and ACT I studies, is Assistant editor of *Stroke*, and is on the editorial boards of *Neurology* and *Journal of Stroke & Cerebrovascular Disease*. Go to Neurology.org for full disclosures.

REFERENCES

1. Markus HS, Larsson SC, Kuker W, et al. Stenting for symptomatic vertebral artery stenosis: the Vertebral Artery Ischaemia Stenting Trial. Neurology 2017;89:1229–1236.
2. Marquardt L, Kuker W, Chandratheva A, Geraghty O, Rothwell PM. Incidence and prognosis of > or = 50% symptomatic vertebral or basilar artery stenosis: prospective population-based study. Brain 2009;132:982–988.
3. Gulli G, Marquardt L, Rothwell PM, Markus HS. Stroke risk after posterior circulation stroke/transient ischemic attack and its relationship to site of vertebrobasilar stenosis: pooled data analysis from prospective studies. Stroke 2013;44:598–604.
4. Chimowitz MI, Lynn MJ, Derdeyn CP, et al. Stenting versus aggressive medical therapy for intracranial arterial stenosis. N Engl J Med 2011;365:993–1003.
5. Wang Y, Wang Y, Zhao X, et al. Clopidogrel with aspirin in acute minor stroke or transient ischemic attack. N Engl J Med 2013;369:11–19.
6. Compter A, van der Worp HB, Schonewille WJ, et al. Stenting versus medical treatment in patients with symptomatic vertebral artery stenosis: a randomised open-label phase 2 trial. Lancet Neurol 2015;14:606–614.
7. Coward LJ, McCabe DJ, Ederle J, Featherstone RL, Clifton A, Brown MM. Long-term outcome after angioplasty and stenting for symptomatic vertebral artery stenosis compared with medical treatment in the Carotid And Vertebral Artery Transluminal Angioplasty Study (CAVATAS): a randomized trial. Stroke 2007;38:1526–1530.
8. Zaidat OO, Fitzsimmons BF, Woodward BK, et al. Effect of a balloon-expandable intracranial stent vs medical therapy on risk of stroke in patients with symptomatic intracranial stenosis: the VISSIT randomized clinical trial. JAMA 2015;313:1240–1248.
9. Compter A, van der Worp HB, Algra A, Kappelle LJ. Risks of stenting in patients with extracranial and intracranial vertebral artery stenosis. Lancet Neurol 2015;14:875.
10. Derdeyn CP, Chimowitz MI, Lynn MJ, et al. Aggressive medical treatment with or without stenting in high-risk patients with intracranial artery stenosis (SAMMPRIS): the final results of a randomised trial. Lancet 2014;383:333–341.

© 2017 American Academy of Neurology. Unauthorized reproduction of this article is prohibited.
